# Use of imaging to assess the activity of hepatic transporters

**DOI:** 10.1080/17425255.2020.1718107

**Published:** 2020-01-23

**Authors:** Irene Hernández Lozano, Oliver Langer

**Affiliations:** a Department of Clinical Pharmacology, Medical University of Vienna, Vienna, Austria; b Division of Nuclear Medicine, Department of Biomedical Imaging and Image-guided Therapy, Medical University of Vienna, Vienna, Austria; c Preclinical Molecular Imaging, AIT Austrian Institute of Technology GmbH, Seibersdorf, Austria

**Keywords:** Hepatic clearance, liver, PET imaging, pharmacokinetics, transporters

## Abstract

**Introduction**: Membrane transporters of the SLC and ABC families are abundantly expressed in the liver, where they control the transfer of drugs/drug metabolites across the sinusoidal and canalicular hepatocyte membranes and play a pivotal role in hepatic drug clearance. Noninvasive imaging methods, such as PET, SPECT or MRI, allow for measuring the activity of hepatic transporters *in vivo*, provided that suitable transporter imaging probes are available.

**Areas covered**: We give an overview of the working principles of imaging-based assessment of hepatic transporter activity. We discuss different currently available PET/SPECT radiotracers and MRI contrast agents and their applications to measure hepatic transporter activity in health and disease. We cover mathematical modeling approaches to obtain quantitative parameters of transporter activity and provide a critical assessment of methodological limitations and challenges associated with this approach.

**Expert opinion**: PET in combination with pharmacokinetic modeling can be potentially applied in drug development to study the distribution of new drug candidates to the liver and their clearance mechanisms. This approach bears potential to mechanistically assess transporter-mediated drug–drug interactions, to assess the influence of disease on hepatic drug disposition and to validate and refine currently available *in vitro-in vivo* extrapolation methods to predict hepatic clearance of drugs.

## 1. Introduction

There is an increasing awareness that drug molecules most often do not cross biological membranes by simple passive diffusion, but require the presence of carrier-mediated processes [1,2]. Herein, membrane transporters belonging to the solute carrier (SLC) and the adenosine triphosphate-binding cassette (ABC) families play a pivotal role. Together, these two transporter families comprise approximately 450 individual members, for one-third of which the exact physiological function still remains unclear. Approximately 30 SLC and ABC transporters have been classified as drug transporters as they are capable of transporting a multitude of different drugs and drug metabolites across cell membranes [3–5]. These transporters are abundantly expressed in clearance organs, such as the liver and the kidney, which together account for the excretion of the majority of drug molecules [6].

**Figure 1.**
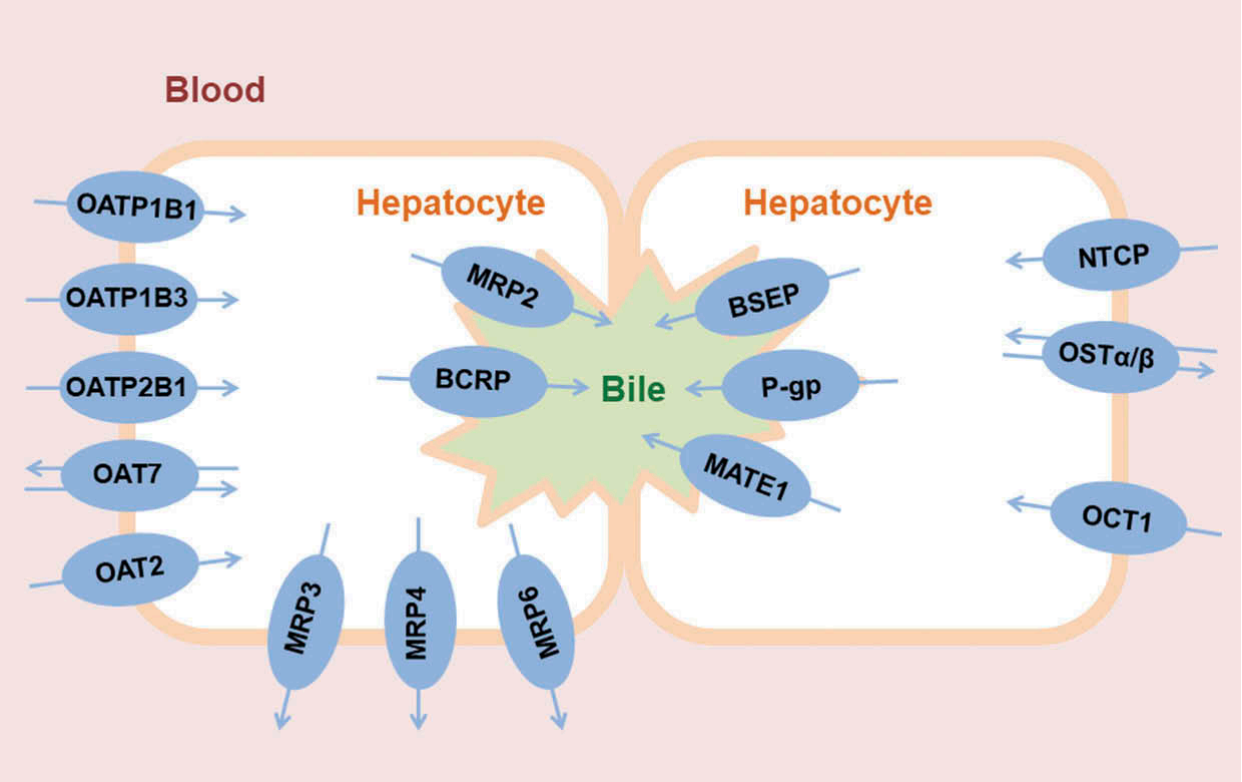
Clinically relevant human drug transporters in the sinusoidal (blood-facing) and canalicular (bile-facing) membranes of hepatocytes. For illustrative purposes, two hepatocytes expressing different transporters are shown in order to depict the functional coupling between sinusoidal and canalicular membrane transporters.

In hepatocytes, several different SLC and ABC transporters are expressed, both in the basolateral membrane facing the sinusoidal blood and in the canalicular membrane, which is the interface to intrahepatic bile canaliculi (Figure 1) [6–8]. In the basolateral membrane, SLC transporters, such as organic anion-transporting polypeptides (OATPs, *SLCO* family) or organic cation transporters (OCTs, *SLC22A* family), mediate the uptake of drugs and drug metabolites from blood into the hepatocyte, while ABC efflux transporters can mediate the backflux of drugs and drug metabolites from the hepatocyte into the blood (i.e. multidrug resistance-associated proteins 3 and 4, MRP3/4, *ABCC3/4*). In the canalicular membrane, ABC transporters, such as P-glycoprotein (P-gp, *ABCB1*), breast cancer resistance protein (BCRP, *ABCG2*) and multidrug resistance-associated protein 2 (MRP2, *ABCC2*), mediate the excretion of drugs and drug metabolites from the hepatocyte into bile. While passive diffusion is believed to play a role for the passage of drugs across the basolateral hepatocyte membrane, the excretion across the canalicular membrane is believed to be mainly transporter-mediated due to the stiffness of the membrane with its high content of sphingolipids and cholesterol [9,10]. There is a functional interplay between basolateral uptake transporters and cytosolic metabolizing enzymes, as the former control the intracellular concentration of drugs available to metabolizing enzymes [11].

Hepatic transporters are of great concern in drug development as they are important sources of pharmacokinetic variability, which may, in turn, lead to inter-individual variability in drug response as well as to drug adverse effects [3]. Important factors which can lead to variability in hepatic transporter activity include genetics, age, disease as well as the concomitant intake of other drugs [12–16]. The latter is referred to as transporter-mediated drug–drug interaction (DDI), in which the simultaneous intake of two drugs which interact with the same drug transporter(s) may change the activity of the transporter(s) as compared to a situation when each drug is taken alone [3,15]. In the liver, such a change in hepatic transporter activity may have important consequences, as it may alter systemic and/or intrahepatic drug concentrations, potentially leading to serious adverse effects. In other cases, drug-induced changes in hepatic transporter activity may lead to altered disposition of physiological transporter substrates, such as bile salts, which can also have severe consequences (i.e. drug-induced cholestasis or liver injury) [8,17]. Regulatory authorities nowadays require the interaction of new drug candidates with drug transporters to be examined in order to assess the risk for the occurrence of transporter-mediated DDIs [18,19].

Based on the recognition that hepatic transporters play a pivotal role in hepatic drug clearance, efforts have been made to incorporate the action of transporters into pharmacokinetic models describing the hepatic clearance of drugs, such as the extended clearance model (ECM) [20]. The ECM provides the mathematical background to identify the rate-determining hepatic clearance step of a drug assuming that the total hepatic intrinsic drug clearance can be expressed as a combination of the individual hepatic elimination processes: passive diffusion and active transport across the sinusoidal membrane into the hepatocytes, potential metabolism of the drug in the cytosol, passive or active backflux into the systemic circulation and efflux clearance at the canalicular membrane of hepatocytes [6,21]. Although it cannot be entirely excluded that lipophilic compounds may penetrate the canalicular membrane of hepatocytes simply by passive diffusion, pharmacokinetic models describing hepatic clearance assume that only active transport of drugs or drug metabolites occurs across this membrane. Moreover, attempts are being made to classify drugs based – among other factors – on the role of transporters in their disposition, such as the biopharmaceutical drug disposition classification system (BDDCS), the extended clearance concept classification system (EC3S), or the extended clearance classification system (ECCS) [22–26]. These models, based on *in vitro* obtained data, predict the relative contributions of elimination pathways in the total clearance of a drug from the system.

Knowledge of the activity of hepatic transporters under different conditions (e.g. during concomitant drug intake or in patients with liver disease) is important to better understand the likelihood for the occurrence of situations, in which drug disposition is altered as compared with the normal population. The most desirable way to assess hepatic transporter activity would be based on the analysis of endogenous or exogenous solutes in biological matrices, which can be straightforwardly sampled in humans, such as blood. As a classical example, serum bilirubin levels can provide information on the activity of hepatic transporters involved in the hepatic handling of bilirubin (i.e. OATP transporters and MRP2) [27]. More recently, considerable efforts have been directed toward identifying a range of different endogenous serum biomarkers, which are indicative of hepatic transporter activity (e.g. coproporphyrin isomers I and III as biomarkers of OATP activity) [28,29]. Physiologically based pharmacokinetic (PBPK) models have attempted to pinpoint the role of individual hepatic transporters in the often complex disposition of these endogenous biomarkers to identify the rate-limiting steps in their disposition [30]. In several cases, however, changes in the activity of hepatic transporters cannot be assessed based on the analysis of serum biomarkers. This is particularly the case when canalicular efflux transporters are involved, as changes in their activities may affect the concentration of drugs or endogenous biomarkers in hepatocytes and in excreted bile, without causing changes in systemic concentrations [20,31]. In these situations, the assessment of transporter activity requires a methodology to quantify intrahepatic concentrations of exogenous or endogenous transporter substrates. In this context, noninvasive imaging methods potentially play a very important role [32,33]. Nuclear imaging methods, such as positron emission tomography (PET) or single-photon emission computed tomography (SPECT) allow for external detection and quantification of the tissue concentration-time profiles of molecules labeled with a positron- or gamma-emitting radionuclide [33,34]. In combination with appropriate radiolabeled probe substrates which are transported by one or several hepatic transporters, PET and SPECT have a great potential to assess the activity of hepatic transporters *in vivo* in humans under various conditions [33]. Moreover, in combination with radiolabeled drugs or drug candidates, these imaging methods can be potentially used to mechanistically assess transporter-mediated DDIs [35] and to validate *in vitro-in vivo* extrapolation (IVIVE) methods of hepatic drug clearance [36,37]. Another imaging method, which is of considerable clinical interest, is magnetic resonance imaging (MRI) in combination with contrast agents which enter and leave hepatocytes mediated by hepatic transporters [33]. Owing to its widespread availability, MRI has the potential to harness hepatic transporter activity as a diagnostic parameter for clinical functional liver imaging.

The aim of the present review is to give an overview of the working principles of imaging-based assessment of hepatic transporter activity with a particular emphasis on PET, to address some important methodological aspects, and to provide an assessment of the potential role of this approach in drug development.

## 2. Positron emission tomography

PET is a nuclear imaging technique that allows visualization and measurement of the concentrations of a molecule labeled with a positron-emitting radionuclide (a so-called radiotracer) in a tissue or organ of interest over time. The emitted positron collides with free electrons in the tissue. This collision annihilates both electron and positron producing two collinear gamma photons that are detected by the PET camera. The main radionuclides used for PET imaging are carbon-11 (^11^C), gallium-68 (^68^Ga), fluorine-18 (^18^F), and zirconium-89 (^89^Zr), which have a radioactive half-life of 20 min, 68 min, 110 min and 3.3 days, respectively, [38]. For the study of drug transporters, PET radiotracers are often based on low molecular-weight drug molecules or analogues thereof [33]. In this context, ^11^C is the preferred PET radionuclide since it allows for radiolabeling without modifying the chemical structure of a drug molecule. On the other hand, ^11^C-labeled radiotracers have a rather narrow clinical applicability due to their short radioactive half-life, which confines their use to specialized imaging centers equipped with a cyclotron and radiochemistry laboratory. Therefore, the synthesis of ^18^F-labeled transporter probe substrates is pursued to come up with radiotracers which can be potentially distributed from a central production site to other hospitals without a radiochemistry facility.

PET is routinely used in clinical oncology as a diagnostic tool, but it is also commonly used to address different research questions in experimental medicine [39–43]. It can for instance be used to assess various aspects of brain and heart function and it has been widely used in drug development [44]. PET is a translational imaging technique, which can be applied both in animals (e.g. mice, rats, non-human primates and pigs) and in humans. PET in combination with radiolabeled drugs can be used to quantitatively describe drug pharmacokinetics *in vivo* at the tissue level, for instance by assessing the rate and extent of distribution of a drug to its pharmacological target tissue or by assessing clearance pathways [34,45–48]. This has been termed ‘pharmacokinetic imaging’ [45,46], which is an upcoming and potentially very powerful approach, even though it presents with certain methodological challenges, which will be discussed in this review article. In general, PET studies are carried out under microdosing conditions (i.e. the mass of unlabeled compound administered with a PET tracer is usually < 100 µg) making it very unlikely that a radiotracer causes any adverse events (e.g. toxicological effects). This additionally offers the advantage that a reduced preclinical toxicology testing package is required for first-in-human applications of novel PET tracers [49]. A comparison of the main characteristics of PET with other imaging techniques is given in Table 1.

**Table 1.** Characteristics of the covered imaging techniques to study hepatic transporter activity.

	PET	SPECT	DCE-MRI
Detection principle	Radioactive decay	Radioactive decay	Nuclear magnetic resonance
Employed nuclei	^11^C (20.4 min), ^18^F (109.8 min)	^99m^Tc (6.1 h), ^123^I (13.3 h)	Gd
Spatial resolution	2 – 8 mm	7 – 10 mm	2 – 5 mm
Temporal resolution	Seconds – minutes	Minutes	Seconds – minutes
Detection sensitivity	10^−11^ – 10^−12^ mol/L	10^−10^ – 10^−11^ mol/L	10^−4^ – 10^−5^ mol/L
Quantification	Quantitative	(Semi)-quantitative	(Semi)-quantitative
Radiation exposure	Yes	Yes	No

## 3. Use of PET to study the activity of hepatic transporters

### 3.1. Requirements for transporter imaging tracers

PET has been applied to study the activity of membrane transporters in animals and humans, mainly at the blood-brain barrier (BBB) and in excretory organs (liver and kidney) [33,50–53]. Typically, radiotracers to study hepatic transporter activity with PET are derived from known drugs or drug metabolites that undergo hepatobiliary excretion or from endogenous molecules such as bile acids [52,54]. In addition, newly developed drugs that are amenable to radiolabeling can be potentially studied with PET to assess their tissue distribution and elimination mechanisms. There is continuous research to identify ideal transporter probe substrates for PET imaging. Some of the main requirements that a radiotracer should fulfill in order to study hepatic transporter activity have been previously discussed [33,52,53]. These include:

- Selectivity for the desired transporter to be studied.
- Lack of metabolism to avoid contamination of the liver PET signal by radiolabeled metabolites.
- Straightforward radiolabeling with a PET radionuclide.
- There must be a substantial, quantifiable difference in the PET signal between situations when the transporter is active and when the transporter is missing or inhibited.
- Sensitivity to measure moderate changes in transporter activity.

As the liver is an important metabolizing organ, the achievement of chemical purity of the PET signal (i.e. absence of radiolabeled metabolites) is particularly challenging. This differs from other organs targeted for imaging, such as the brain, from which radiolabeled metabolites are often excluded due to the presence of the BBB. One possible approach to achieve the metabolic stability of a hepatic transporter PET tracer is to radiolabel drug metabolites, which do not undergo further metabolism, as exemplified by the major metabolite of the cyclooxygenase-2 inhibitor celecoxib [^11^C]SC-62,807 [55]. However, depending on the localization of the transporter of interest, metabolic stability may not always be a stringent requirement. For instance, in order to assess the activity of basolateral hepatic uptake transporters only data comprising the first few minutes after radiotracer injection are usually considered, during which the exchange of radiotracer between blood and the hepatocyte occurs and during which radiotracer metabolism is often negligible [56–58]. On the other hand, if the main interest is to study a biliary efflux transporter, the radiotracer must be metabolically stable and it is desirable that it has good permeability across the sinusoidal membrane. Another very challenging requirement in the development of hepatic transporter imaging tracers is selectivity for the transporter of interest. Given the wide and largely overlapping substrate spectrum of most hepatic transporters, selectivity for one single transporter is in fact very difficult to achieve. While compartmental modeling approaches allow for separately assessing the effects of basolateral uptake, basolateral efflux and canalicular efflux transporters, they do not allow for discriminating the contribution of different basolateral uptake transporters (e.g. OATP1B1, OATP1B3 and OATP2B1) or different canalicular efflux transporters (e.g. MRP2 and BCRP) to the hepatic handling of a transporter imaging tracer. To achieve a high magnitude of the transporter-specific PET signal, the transporter of interest would ideally account for the rate-limiting step in the overall hepatic clearance of the radiotracer, i.e. in absence of the transporter hepatic clearance would be drastically reduced. It is important to not confound a high magnitude of the transporter-specific PET signal with a good sensitivity to measure moderate changes in transporter activity. For instance, some radiotracers are so efficiently transported by OATPs that their hepatic uptake clearance approaches the hepatic blood flow (e.g. [^11^C]rosuvastatin) [37]. Even though such radiotracers may produce a large change in PET signal when the OATP transporters are knocked out or completely inhibited, they may lack the sensitivity to measure small alterations in transporter activity. This is because in cases of moderately reduced OATP abundance/activity, remaining transport capacity may still be sufficient for the radiotracer to display blood flow-limited hepatic uptake. Therefore, radiotracers which are less efficiently transported by OATPs may display a better sensitivity to measure OATP activity [58,59]. Taken together, the development of an effective radiotracer to measure hepatic transporter activity is a very challenging and far from trivial task [35].

### 3.2. Measuring the activity of hepatic drug transporters with PET

Table 2 includes a list of PET tracers that have been used to measure hepatic transporter activity in animals and humans and the transporters that have been studied. Some of these radiotracers have so far only been applied in animals, mostly rodents. Human translation can be a challenge as for some hepatic transporters (notably members of the *SLCO* family) no direct rodent orthologues of human transporters exist.

**Table 2.** PET radiotracers to study the activity of hepatic transporters.

PET radiotracer	Hepatic transporters	Species	Application	Ref
[^11^C]*N*-acetyl-cysteinyl-leukotriene E_4_	OATPs, MRP2	Rats, Monkeys	Probe validation	[74]
(15*R*)-[^11^C]TIC-Me	OATPs, MRP2	Rats, Humans	Probe validation	[57,63]
[^11^C]SC-62,807	OATP1B1/3, BCRP	Mice, Rats	Probe validation	[55,73]
[^11^C]Telmisartan	OATP1B3	Rats, Humans	Mechanistic assessment of hepatic clearance; DDI study with rifampicin; assessment of dose dependency of hepatic disposition	[56,64–66]
[^11^C]Cholylsarcosine	OATPs, NTCP, BSEP	Pigs, Humans	Probe validation and evaluation of altered hepatic transporter activity in liver disease	[87–90]
[^11^C]Dehydropravastatin	OATP1B1/3, MRP2	Rats, Humans	Probe validation	[67–69]
[^11^C]Rhodamine-123	OCT1, P-gp	Mice, Rats	Probe validation	[79]
[^11^C]Glyburide	OATPs	Mice, Baboons	Mechanistic assessment of hepatic clearance; DDI study with rifampicin and cyclosporine A	[70]
[^11^C]Metformin	OCT1, MATE1	Mice, Humans	Assessment of the effect of genetic polymorphisms or disease on liver distribution as pharmacological target tissue	[75–78,96]
[^11^C]Rosuvastatin	OATPs, NTCP, BCRP, P-gp, MRP2	Rats, Humans	Mechanistic assessment of hepatic clearance; DDI study with rifampicin and cyclosporine A; validation of IVIVE	[37,94]
[^11^C]Erlotinib	OATP2B1, BCRP, P-gp,	Mice, Rats, Humans	Mechanistic assessment of hepatic clearance; DDI study with rifampicin; assessment of dose dependency of hepatic disposition	[60,61,97,98]
[^18^F]Pitavastatin	OATP1B1/3, BCRP, MRP2	Rats	Mechanistic assessment of hepatic clearance; DDI study with rifampicin	[58,71]
[^18^F]PTV-F1	OATP1B1/3	Rats	Probe validation	[59,72]
3β-[^18^F]Fluorocholic acid	OATPs, NTCP, BSEP	Mice	Probe validation and evaluation of altered hepatic transporter activity in mouse models of liver disease	[92,93]
[^11^C]Sulpiride	OCT1	Mice, Humans	Mechanistic assessment of hepatic clearance; assessment of dose dependency of hepatic disposition	[80]
[^11^C]Tariquidar	P-gp, BCRP	Mice, Humans	Probe validation; assessment of dose dependency of hepatic disposition	[84]

Most transporter imaging work in the liver has been done with respect to OATP transporters [62]. OATPs are key transporters mediating the uptake of drugs from blood into hepatocytes and they have a broad substrate specificity. The activity of hepatic OATPs has been studied with PET in rodents, baboons and humans with radiotracers such as (15*R*)-16-m-[^11^C]tolyl-17,18,19,20-tetranorisocarbacyclin methyl ester ((15*R*)-[^11^C]TIC-Me) [57,63], [^11^C]telmisartan [56,64–66], [^11^C]dehydropravastatin [67–69] or [^11^C]glyburide [70]. Moreover, studies in rats with [^18^F]pitavastatin and its analog [^18^F]PTV-F1 indicated that these two radiotracers are potentially suitable for a more sensitive detection of changes in OATP transporter activity as compared to previously developed OATP imaging tracers, as their hepatic uptake clearance is not rate-limited by hepatic blood flow [58,59,71,72]. Most of the radiotracers used to study hepatic OATP transporters are not selective for OATP subtypes (i.e. OATP1B1 and OATP1B3). In addition, most of them are also substrates of canalicular membrane transporters belonging to the ABC transporter family. For instance, [^11^C]SC-62,807, which is a substrate of OATP1B1 and OATP1B3, was shown to be also a substrate of BCRP and could be used to study the activity of BCRP in the mouse liver [55,73]. Other radiotracers, such as [^11^C]dehydropravastatin [67–69], (15*R*)-[^11^C]TIC-Me [57,63] and [^11^C]*N*-acetyl-cysteinyl-leukotriene E_4_ [74], have been shown to be not only substrates of OATP transporters but also to be useful for the study of canalicular MRP2 activity. Other PET tracers have been used to study organic cation transporters of the *SLC22A* family, including [^11^C]metformin [75–78], [^11^C]rhodamine-123 [79] and [^11^C]sulpiride [80]. The radiolabeled P-gp inhibitor [^11^C]tariquidar has been developed to measure P-gp and BCRP activity at the BBB in mice and humans [81–83]. Recently, we performed studies with [^11^C]tariquidar in mice and humans which suggested that this radiotracer might be also useful to measure P-gp and BCRP activity in the canalicular membrane of hepatocytes without a confounding effect of basolateral uptake transporters [84].

### 3.3. Measuring the activity of bile acid transporters with PET

Bile acids are taken up at the basolateral membrane of hepatocytes by the sodium (Na^+^) taurocholate co-transporting polypeptide (NTCP, *SLC10A1*) and by OATPs while their secretion at the canalicular membrane is mediated by the bile salt export pump (BSEP, *ABCB11*) and by MRP2 [85]. Inhibition of canalicular efflux transporters by certain drugs can lead to hepatic accumulation of bile acids which might become toxic to the liver and eventually cause liver damage such as drug-induced liver injury [86]. The primary bile acid transporters in hepatocytes (NTCP and BSEP) are – as opposed to some other hepatic transporters – very selective in their substrate spectrum and currently available drug-derived radiotracers are usually not recognized by these transporters. Consequently, radiolabeled bile acids have been developed in order to visualize and quantify hepatobiliary transporter activity relevant for hepatic bile salt handling. This was not trivial in terms of synthetic chemistry, as radiolabeling of bile acids with positron-emitting radionuclides cannot be straightforwardly achieved. Pioneering work has been done with [^11^C]cholylsarcosine, the *N*-[^11^C]methyl analogue of the endogenous bile acid conjugate cholylglycine, which was used to visualize and quantify the *in vivo* kinetics of hepatobiliary secretion of bile acids in pigs [87,88]. In later studies, [^11^C]cholylsarcosine PET was applied in human healthy volunteers as well as in patients with cholestatic liver disease [89,90]. Reduced biliary secretion was found in patients with cholestatic liver disease, supporting that radiolabeled bile salt derivatives can be used to quantify changes in hepatobiliary transporter activity caused by disease [89]. In addition, a ^18^F-labeled analogue of cholylglycine, i.e. *N*-(4-[^18^F]fluorobenzyl)cholylglycine, has been synthesized to study the enterohepatic circulation of bile acids in rats, for which the longer radioactive half-life of ^18^F proved beneficial [91]. Moreover, a ^18^F-labeled analogue of unconjugated cholic acid, i.e. 3β-[^18^F]fluorocholic acid ([^18^F]FCA), was synthesized and shown to be *in vitro* a substrate of OATP1B1, OATP1B3, NTCP, BSEP, and MRP2 [92]. *In vivo* studies in mice pre-treated with the NTCP and the BSEP inhibitor bosentan or with rifampicin (prototypical OATP inhibitor) revealed significant changes in the pharmacokinetics of [^18^F]FCA when these inhibitors were administered [92]. Recently, this radiotracer has also been applied in different mouse models of liver disease [93]. Taken together, the studies with radiolabeled bile acids or their analogues highlight the great potential of imaging-based evaluation of bile acid transporter activity in liver disease and during drug development in order to identify new drug candidates that might inhibit these transporters.

### 3.4. PET to assess transporter-mediated DDIs

One potentially important application of PET to study hepatic membrane transporter activity is the mechanistic assessment of transporter-mediated DDIs [35,53]. Provided that a drug candidate is a potential victim of transporter-mediated DDIs, one approach is to radiolabel the suspected victim drug for PET and to assess the influence of prototypical transporter inhibitors (e.g. rifampicin for OATPs) or other drugs, which are expected to be co-administered with the victim drug in the clinic, on the liver pharmacokinetics of the radiolabeled victim drug. The great advantage of this approach is that the radiolabeled victim drug can be administered at microdoses (< 100 µg) so that no safety concerns are expected to be encountered, when the systemic exposure and tissue distribution of the victim drug changes due to transporter inhibition. This is a potentially very powerful approach as it allows, provided that a suitable pharmacokinetic model is applied, to measure the rate constants or clearances for transfer of the radiolabeled victim drug across hepatocyte membranes and to elucidate the involvement of hepatic transporters expressed in these membranes. A limitation of this approach is the workload and cost associated with the radiolabeling of a drug candidate and obtaining approval by competent authorities for application in humans.

Provided that a drug candidate is a suspected perpetrator of transporter-mediated DDIs, PET with a prototypical transporter substrate radiotracer, which displays ideal characteristics to measure the activity of one or several hepatic transporters (Table 2), can be used in combination with administration of the unlabeled drug candidate. A challenge of this approach is to extrapolate any observed changes in the liver pharmacokinetics of the prototypical transporter substrate radiotracer caused by transporter inhibition to actual victim drugs encountered in the clinic. Next to its potential for assessing transporter-mediated DDIs in drug development, this second approach has been used in several studies to validate newly developed radiotracers for transporter imaging. In a few studies, rifampicin was used to inhibit hepatic OATP transporter activity to prove the ability of some radiotracers to assess hepatic OATPs. For instance, reduction in the liver uptake and biliary efflux clearances of (15*R*)-[^11^C]TIC-Me was observed after oral administration of rifampicin in human healthy volunteers [57]. Similarly, liver uptake and biliary efflux clearances of [^11^C]dehydropravastatin were significantly reduced after rifampicin administration in both rats and humans indicating the involvement of OATP transporters and MRP2 in the hepatic disposition of this radiotracer [68,69]. In another study, co-injection of rifampicin or unlabeled telmisartan with [^11^C]telmisartan decreased the hepatic uptake clearance of [^11^C]telmisartan in rats with no effect on biliary efflux clearance, suggesting that the hepatic uptake of [^11^C]telmisartan in rats was mainly dependent on OATP transporters [56]. However, pre-treatment with unlabeled telmisartan in humans delayed the systemic elimination of [^11^C]telmisartan but did not change the hepatic uptake clearance of the radiotracer indicating that in humans other mechanisms than the saturation of OATP uptake transporters accounted for non-linearity in [^11^C]telmisartan pharmacokinetics [66]. One study in baboons assessed the influence of rifampicin or cyclosporine A on the whole-body distribution of the radiolabeled oral antidiabetic drug [^11^C]glyburide [70]. Both inhibitors caused a significant reduction in [^11^C]glyburide liver exposure as well as an increase in the plasma area under the curve (AUC), which pointed to a major contribution of OATP transporters to [^11^C]glyburide liver uptake. In one study with the radiolabeled statin drug [^11^C]rosuvastatin in rats, the presence of rifampicin caused a pronounced increase in the blood AUC as well as a reduction in the liver uptake clearance of [^11^C]rosuvastatin, which was attributed to an inhibition of OATP uptake transporters in rats [94]. Additionally, in one study in healthy human volunteers, intravenous infusion of cyclosporine A caused an increase in the [^11^C]rosuvastatin blood AUC and also inhibited the liver uptake clearance in three out of four subjects [37]. These results supported an involvement of OATP transporters in the uptake of [^11^C]rosuvastatin from the blood into the liver in humans. Studies in mice with the radiolabeled antidiabetic drug [^11^C]metformin indicated delayed washout and enhanced accumulation of radioactivity in the liver after pretreatment with pyrimethamine or cimetidine, two prototypical inhibitors of the multidrug and toxin extrusion protein 1 (MATE1, *SLC47A1*). Pretreatment with pyrimethamine did not reduce the liver uptake clearance of [^11^C]metformin, suggesting lack of *in vivo* potency of pyrimethamine to inhibit metformin uptake transporters in the liver (i.e. OCT1, *SLC22A1*) [78]. Studies in mice knocked out for *Slc22a1* and *Slc22a2* resulted in a reduced liver exposure to [^11^C]metformin demonstrating the important role of OCT1 in mediating the liver distribution of [^11^C]metformin [77]. Subsequent studies have assessed the hepatic disposition of [^11^C]metformin in humans [75,95,96]. In one study it was shown that [^11^C]metformin exposure in the liver expressed as volume of distribution (*V*_T_) was significantly lower in carriers of p.M420del and p.R61C variants in the *SLC22A1* gene after both oral and intravenous administration of the PET tracer, which supported the notion that genetic *SLC22A1* variants may affect metformin response.

An example of a DDI study with PET was with the radiolabeled tyrosine kinase inhibitor [^11^C]erlotinib, whose hepatic disposition was studied in healthy human volunteers, mice, and rats without and with pre-treatment with rifampicin. In mice and rats, rifampicin caused a pronounced reduction in the rate constant for the uptake of [^11^C]erlotinib from the blood into the liver, while a similar, but markedly less pronounced decrease was observed in humans. These results indicated that rifampicin-inhibitable transporters, possibly OATP2B1 (*SLCO2B1*), contributed to the distribution of [^11^C]erlotinib from the blood into the liver [97,98].

## 4. Other imaging techniques to study transporter activity in the liver

Other imaging techniques, such as SPECT or MRI, have also been used to study hepatic transporter activity *in vivo* [51,62,99]. With technological advances in SPECT methodology, fully quantitative data on the tissue pharmacokinetics of SPECT tracers can be obtained, which can be subjected to similar pharmacokinetic modeling approaches as employed for PET tracers. An advantage of SPECT over PET is its widespread clinical availability and the need for less complex research infrastructure to perform SPECT radiolabeling. Moreover, there is a broad range of commercially available diagnostic SPECT tracers. Some of these interact with hepatic transporters and can, therefore, be potentially repurposed for hepatic transporter imaging, such as [^99m^Tc]mebrofenin. MRI produces high-resolution images of anatomy and physiological processes. In combination with contrast agents, dynamic contrast-enhanced (DCE) MRI has been applied to study hepatic transporter activity. However, compared to PET and SPECT, DCE-MRI lacks sensitivity, requiring the administration of high doses of the contrast agents, and absolute quantification of the liver concentrations of these contrast agents is challenging [100] (Table 1). Table 3 summarizes SPECT tracers and MRI contrast agents, which have been applied to study transporter activity in the liver.

**Table 3.** SPECT and MR imaging agents used to study the activity of hepatic transporters.

Imaging agent		Hepatic transporters	Species	Application	Ref
	**SPECT**				
[^99m^Tc]Mebrofenin		OATP1B1/3, MRP2, MRP3	Mice, rats, humans	Mechanistic assessment of hepatic clearance; assessment of the effect of genetic polymorphisms or disease on hepatic transporter activity	[106–112]
[^99m^Tc]PMT		OATP1B1/3, P-gp, MRP2	Rats, rabbits, humans	Probe validation	[116]
[^111^In]EOB-DTPA		OATPs	Mice	Probe validation	[117]
[^99m^Tc]DTPA-CDCA		OATP1B1/3, MRP2	Mice	Probe validation	[121]
[^99m^Tc]DTPA-CA		OATP1B1/3, MRP2	Mice	Probe validation	[121]
[^131^I]NP-59		OATP1B1/3, BCRP	Mice	Probe validation	[120]
	**MRI**				
Gadoxetate		OATPs, MRP2	Mice, rats, humans	Mechanistic assessment of hepatic clearance; assessment of the effect of genetic polymorphisms or disease on hepatic transporter activity	[101,102,124–132]
BOPTA		OATPs, MRP2	Perfused rat livers	Mechanistic assessment of hepatic clearance	[133–137]

### 4.1. SPECT tracers to study liver transporters

Commonly used SPECT radionuclides are technetium-99m (^99m^Tc, half-life: 6.1 h), iodine-123 (^123^I, half-life: 13.3 h) and indium-111 (^111^In, half-life: 2.8 days). [^99m^Tc]Mebrofenin has been widely used to provide an early diagnosis in hepatobiliary disease [103–105] and has also been used to study hepatic transporter activity. *In vitro* studies have confirmed that [^99m^Tc]mebrofenin is taken up into hepatocytes by OATP1B1 and OATP1B3 [106–108], while biliary excretion is mediated by MRP2 and sinusoidal backflux by MRP3 [106,108]. *In vivo* studies in HsdAMC:TR-*Abcc2* mutant rats, which lack functional MRP2, showed that the biliary excretion of [^99m^Tc]mebrofenin was impaired, highlighting the contribution of MRP2 to the hepatobiliary excretion of [^99m^Tc]mebrofenin [109]. One study in mice showed decreased [^99m^Tc]mebrofenin liver AUC in *Slco1a/1b* knockout mice, while in *Abcc2* knockout mice there was an increase in the liver AUC and a decrease in the gall bladder and intestine AUC [110]. [^99m^Tc]Mebrofenin has been used in humans for liver transporter imaging [106,111,112]. One study in healthy volunteers assessed the effect of ritonavir (MRP2 and OATP inhibitor) on [^99m^Tc]mebrofenin liver distribution. Ritonavir was found to lead to an increase in the systemic [^99m^Tc]mebrofenin exposure but no decrease was found in its biliary excretion [111]. Another study with [^99m^Tc]mebrofenin found in patients with nonalcoholic steatohepatitis (NASH) an increase in the systemic and the hepatic exposure of [^99m^Tc]mebrofenin as compared with healthy subjects due to a decrease in biliary clearance, suggesting that hepatic MRP2 activity is impaired in NASH [112]. Taken together, these data support that [^99m^Tc]mebrofenin SPECT can be used to measure hepatocyte OATP1B1, OATP1B3 and MRP2 activity *in vivo*.

Another SPECT tracer which has been used to diagnose hepatobiliary diseases is [^99m^Tc]*N*-pyridoxil-5-methyltryptophan ([^99m^Tc]PMT) [113–115]. One *in vitro* study showed that [^99m^Tc]PMT was mainly transported by OATP1B1 and OATP1B3 [116]; however, the *in vivo* transport mechanisms for uptake and efflux of [^99m^Tc]PMT in hepatocytes have not yet been elucidated. [^111^In]Indium-ethoxybenzyl-diethylenetriamine-pentaacetic acid ([^111^In]EOB-DTPA) was derived from the MRI contrast agent Gd-EOB-DTPA (gadoxetate) and was evaluated in mice confirming higher uptake in NTCP overexpressing tumor xenografts [117]. Uptake of radioactivity in the mouse liver suggested that [^111^In]EOB-DTPA may be used to assess the activity of hepatic OATP transporters. Another study showed that [^131^I]6-β-iodomethyl-19-norcholesterol ([^131^I]NP-59), a cholesterol analog which is used to localize adrenal cortical lesions and which is mainly excreted *via* the hepatobiliary route [118,119], was transported by OATP1B1, OATP1B3 and BCRP, suggesting a potential applicability for hepatic transporter imaging [120]. As for PET imaging, bile acid analogs have also been radiolabeled with SPECT radionuclides in order to study bile acid transporter activity. One example is a study performed with ^99m^Tc-labeled chenodeoxycholic acid ([^99m^Tc]DTPA-CDCA) and cholic acid ([^99m^Tc]DTPA-CA) [121]. *In vitro* experiments indicated transport of both tracers by OATP1B1, OATP1B3 and MRP2, but not by NTCP and BSEP. Moreover, *in vitro* results were confirmed *in vivo* in mice treated with rifampicin, which revealed reduced liver, gall bladder and intestinal AUCs, suggesting inhibition of transport of [^99m^Tc]DTPA-CDCA and [^99m^Tc]DTPA-CA by OATPs and MRP2.

### 4.2. MRI contrast agents to study liver transporters

MRI is used in clinical routine examinations for the detection and evaluation of focal liver lesions [122]. Several MRI contrast agents have been shown to be transported by hepatic uptake and efflux transporters. Among these, gadoxetate (Gd-EOB-DTPA) has been the most commonly investigated. *In vitro* studies in *Xenopus laevis* oocytes and in HEK 293 cells found that gadoxetate is transported by OATP1B1 and OATP1B3 and is also a weak substrate of NTCP [123–125]. Additional *in vitro* studies reported that gadoxetate was also transported by MRP2, which mediated the biliary excretion of gadoxetate in rats [124]. DCE-MRI with gadoxetate has been used in order to assess changes in hepatic transporter expression and localization related to liver disease. Studies in congestive rat livers [126], a rat model of advanced liver fibrosis [127] and a rat model of liver cirrhosis [128] have shown correlations between the reduced expression of rat OATP1A1 (*Slco1a1*) and/or MRP2 and changes in contrast enhancement in the liver. A study in a mouse model for type 2 diabetes [129] found reduced protein expression of OATP1A1 and OATP1B2 (*Slco1b2)* in diabetic mice, which was mirrored by reduced liver uptake of gadoxetate, suggesting a potential clinical applicability of DCE-MRI with gadoxetate in human diabetic patients. In hepatocellular carcinoma patients, one study showed an increased accumulation of gadoxetate in the tumor cells due to an increase in OATP1B1 and OATP1B3 expression and a decrease in MRP2 expression relative to healthy liver tissue [130]. DCE-MRI with gadoxetate has also been used in DDI studies in mice and rats, in which novel MRI quantification methods showed a reduction in the uptake and efflux rates of gadoxetate after treatment with rifampicin [131,132].

Another MRI contrast agent that has been used to elucidate transporter activity in the liver is BOPTA (gadolinium benzyl-oxypropionictertraacetate). *In vitro* experiments indicated that BOPTA is transported by rodent OATP1A1, OATP1A2 (*Slco1a2*), OATP1B2 and MRP2, and *in situ* perfusion of rat livers confirmed that intrahepatic concentrations of BOPTA are controlled by both OATP transporters and by MRP2 [133]. Some other studies have been performed using BOPTA in perfused rat livers in order to study the pharmacokinetics of this contrast agent in health and disease as well as to assess transporter-mediated DDIs [134–137].

## 5. Quantitative analysis of imaging data to assess hepatic transporter activity

Mathematical models can be used in order to quantitatively describe the hepatic disposition of a PET tracer and to obtain parameters that can be directly related to membrane transporter activity. These mathematical methods use the time–activity curves (TACs) of different hepatic regions of interest obtained from the PET images and the blood or plasma TAC, which is usually obtained from blood samples collected during the PET scan. A graphical analysis method called integration plot has been used to determine quantitative pharmacokinetic parameters, i.e. the hepatic uptake clearance, which is calculated from the blood TAC and the cumulative amount of radioactivity taken up by the liver (considering usually only data acquired during the first few minutes after radiotracer injection), and the biliary efflux clearance, calculated from the liver TAC and the cumulative amount of radioactivity excreted into the bile [57]. These clearances correspond to the slope of the linear part of the integration plots. Although this mathematical method has been applied in several studies to assess transporter activity and the involvement of transporters in the hepatic clearance of radiolabeled drugs [57,69,78], it does not provide a complete picture of the hepatic disposition of a radiotracer. Integration plot analysis does not allow to assess the backflux of a radiotracer through the basolateral membrane of hepatocytes into blood, unless the cumulative amount of radiotracer excreted from hepatocytes into the extracellular space is known, which, however, cannot be derived from the PET data. Basolateral backflux might be mediated by basolateral efflux transporters and make a major contribution to total hepatic clearance of a given radiotracer, as in the case of e.g. [^11^C]rosuvastatin [37]. In addition, in several cases failure to identify a linear phase in the integration plot (in particular for estimation of biliary efflux clearance), makes it often difficult to objectively derive the clearance values from the plot.

**Figure 2.**
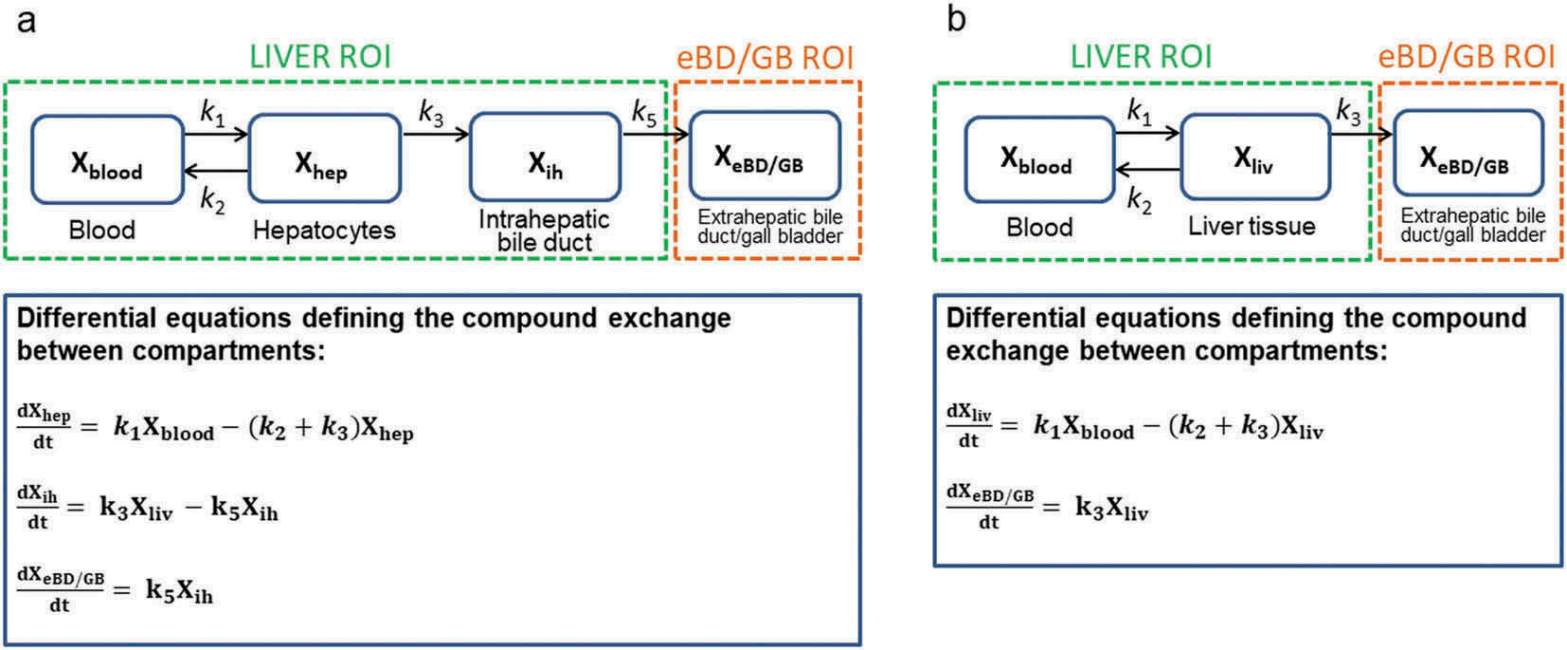
Pharmacokinetic models developed to study the hepatobiliary disposition of [^11^C]erlotinib. (a) Four compartment model developed from the combination of two previously developed models [60,89]. This model includes two main regions of interest (ROIs): the liver and the extrahepatic bile duct and gall bladder (eBD/GB). The liver ROI includes compartments representing the amount of radioactivity in the blood fraction in the liver sinusoids used as the model input function (X_blood_, 0.25% of the total liver volume), in the hepatocytes (X_hep_) and in the intrahepatic bile (X_ih_, 0.32% of the total liver volume). The extrahepatic ROI compartment represents the amount of radioactivity in the visible part of the extrahepatic bile duct and the gall bladder. The kinetic parameters define the exchange rate of radioactivity between blood and hepatocytes (*k*_1_ and *k*_2_), from hepatocytes to the intrahepatic bile duct (*k*_3_), or from the intrahepatic bile duct to the extrahepatic bile duct and gall bladder (*k*_5_). (b) The three compartment model includes compartments representing the blood in the liver sinusoids (used as the model input function), liver tissue (combination of the hepatocytes and the intrahepatic bile duct from the four compartment model) and excreted bile out of the liver ROI. The kinetic parameters define the exchange rate of radioactivity between the blood and the liver (*k*_1_ and *k*_2_) or from the liver to the excreted bile (*k*_3_). The differential equations depicted under the kinetic models are implemented to fit the amount of radiotracer in each compartment to the obtained PET TACs and obtain the estimates of the kinetic parameters. In the equations, X represents the amount of radiotracer in the compartment and *k*s are the respective rate constants. Note that *k*_1_ represents the basolateral uptake rate and does not discriminate between active or passive uptake mechanisms. According to PET pharmacokinetic modeling, the parameter *K*_1_ is perfusion-dependent and can be related to blood flow (Q) as: *K*_1_ = E x Q; where E represents the unidirectional first-pass extraction ratio [140]. In the present model, *k*_1_ can be expressed by means of *K*_1_ as *k*_1_ = *K*_1_ x (V_liver_/V_blood_), where V_liver_ corresponds to the volume of hepatocytes in (a) and the volume of liver tissue in (b), and V_blood_ corresponds to the volume of blood in the hepatic sinusoids. Therefore, the uptake rate constant *k*_1_ can be expressed in terms of perfusion as: *k*_1_ = (E x Q x V_liver_)/V_blood._

An alternative approach to quantitatively analyze the TACs obtained from the PET images is to implement compartmental PBPK models. In PBPK models, the body is described as different interconnected compartments that represent a specific tissue or organ of interest. The amount of radioactivity in each compartment is mathematically defined by an ordinary differential equation which describes the rate of change of the radiotracer in the tissue (Figure 2). Kinetic models for PET are frequently composed of up to three compartments, to which the blood or plasma curve (typically obtained from collected arterial blood samples) serves as the model input function [138]. Usually the model input function is corrected for radiolabeled metabolites by chromatographic analysis of plasma samples (i.e. by high-performance liquid chromatography). Alternatively, the arterial input function may be derived from the PET images by placing a region of interest into the hepatic aorta [139]. Image-derived input functions, however, are only applicable for radiotracers which are not extensively metabolized, as metabolite correction is not straightforward. In order to obtain the pharmacokinetic parameters that describe the transfer of radioactivity between the different specified compartments, the model equations are usually implemented in specific pharmacokinetic analysis software such as NONMEM or SAAM II. In addition, custom-written scripts have been developed in programming languages such as MATLAB and there is also freely available software such as *iFit*, in which established liver pharmacokinetic models are implemented to fit liver PET data [88]. The obtained kinetic parameters (i.e. rate constants for transfer of radiotracer between different liver compartments) can be transformed into hepatic clearances (e.g. basolateral uptake clearance, basolateral backflux clearance or canalicular efflux clearance), which can be directly related to transporter activity *in vivo*. Several pharmacokinetic models have been proposed to describe the hepatobiliary disposition of intravenously administered PET tracers and have been applied to the animal (e.g. mice and pigs) and human data. For instance, the model developed to study the hepatobiliary distribution of [^11^C]rosuvastatin in rats was a five compartment model, which included not only liver and intestine (excreted bile) data but also kidney and metabolite compartments [94]. In this model, the disposition of [^11^C]rosuvastatin in the blood compartment was modeled, i.e. the blood TAC did not serve as an input function to the model. With this model, relevant parameters such as the hepatic uptake clearance and the sinusoidal and biliary efflux clearances could be obtained and the major elimination routes of [^11^C]rosuvastatin could be described. A simpler three-compartment model was applied to human data, in which the measured blood curve served as a model input function, to study [^11^C]rosuvastatin disposition and the model-derived total hepatic plasma clearance was found to be in the same range as previously reported values using non-PET data [37]. Compartmental modeling analysis revealed for the first time that the major efflux route of rosuvastatin from hepatocytes is sinusoidal backflux rather than biliary efflux [37,94]. This highlights the particular strengths of PET imaging, as no other currently available method is able to estimate sinusoidal backflux clearance of drugs *in vivo*.

**Figure 3.**
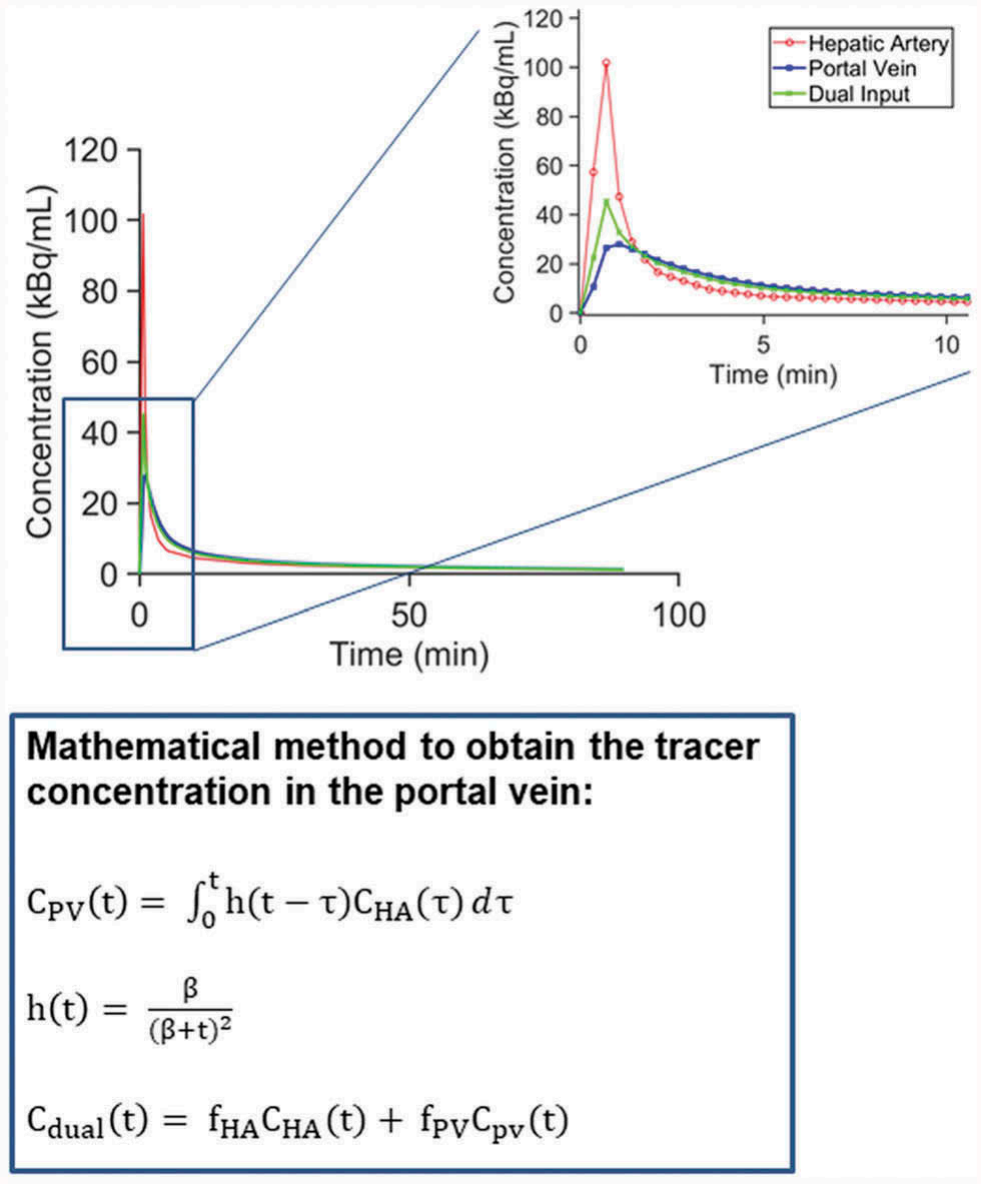
Representation of [^11^C]erlotinib radioactivity concentration in the radial artery (assumed to be equal to the concentration in the hepatic artery), in the mathematically-derived portal vein and in the estimated dual input function in one representative subject. The enlarged graph section shows only the first 10 min of the PET scan duration for which the difference in concentration is larger between arterial and venous hepatic blood. The equations depicted below the graph show the mathematical method implemented to estimate the radiotracer concentration in the portal vein. The concentration in the portal vein along time, C_PV_(t), can be calculated as the convolution integral between the concentration in the hepatic artery, C_HA_(t), and the impulse-response function, h(t) as described in the first equation. This impulse-response function (second equation) is mainly characterized by the parameter β which determines the mean transit time for the passage of radiotracer from the intestine to the portal vein. Finally, as indicated in the third equation, the total concentration in the liver sinusoids (dual input function, C_dual_(t)) can be represented as a flow-weighted function which takes into account the hepatic artery flow fraction (f_HA_, ~0.25) and the portal vein flow fraction (f_PV_, ~0.75).

However, application of these mathematical models (including integration plot analysis) to the liver is complicated by the fact that the liver receives a dual blood supply, from the hepatic artery (25% of the total blood input) and from the portal vein (75%). Moreover, because of the transfer of radiotracer through the splanchnic circulation, the radiotracer concentration in the portal vein is initially delayed and dispersed compared to its time-course in the hepatic artery [141]. Therefore, considering only the radiotracer concentration in the sampled or image-derived arterial blood is not accurate. Some studies suggested that, assuming no loss of radiotracer in the splanchnic circulation, the use of a single arterial input with a time delay would suffice to represent the blood input to the liver [142]. However, other studies showed that a dual blood input function, including the radiotracer input from both vessels, is needed to obtain unbiased kinetic parameter estimates and that the use of solely an arterial input underestimates the rapid blood-tissue exchange [143]. Since the blood from the portal vein cannot be sampled in humans and its TAC is not easily derived from the PET images, some pharmacokinetic models have adopted a mathematical approach to consider the blood input from both, the hepatic artery and the portal vein [88,89]. This approach, which was validated in pigs (from which the portal vein blood can be sampled), mathematically estimates the portal vein TAC mainly from the sampled arterial blood and a parameter (β), which describes the mean transit time of the radiotracer from the intestinal arteries to the portal vein [141]. For a given radiotracer, the β value can either be experimentally derived in pigs and applied to humans (assuming conservation of the β value across species) [88,141] or it may be included as a fitting parameter into the model [141,144] (Figure 3). Although this mathematical approach to obtain the radiotracer concentration in the portal vein has been validated in pigs, further validation in humans would be needed as the β parameter may vary between species. Since the blood from the portal vein cannot be sampled in humans, PET in combination with other higher-resolution imaging methods might be used in order to obtain accurate image-derived portal vein TACs and validate the mathematical method. Based on the estimated portal vein TAC and the sampled arterial TAC, a flow-weighted dual input function can be generated as the model input function (Figure 3). A three compartment model implementing the dual-input function (based on the β parameter obtained from pigs) was developed to measure the hepatobiliary kinetics of [^11^C]cholylsarcosine in healthy human volunteers and in patients with various cholestatic disorders [89]. This model helped to quantify the separate transport steps in the hepatobiliary secretion of [^11^C]cholylsarcosine. In this model, the rate constant describing the flow of [^11^C]cholylsarchosine from hepatocytes to bile (*k*_3_) was calculated based on the intrahepatic excreted bile, which is assumed to account for only 0.32% of the total radioactivity observed in the liver region of interest and which is not visible on the PET images. Although the radiotracer concentrations in the common hepatic duct were also measured, this model did not include a compartment representing the extrahepatic excreted bile. Based on this model, we developed a similar four compartment model to describe the hepatobiliary kinetics of [^11^C]erlotinib in humans [144], in which an additional compartment describing the amount of radiotracer in the extrahepatic bile duct and in the gall bladder was included. The parameter *k*_5_ represented the transfer of radioactivity from the intrahepatic bile duct to the extrahepatic excreted bile (Figure 2(a)). As a simplification of this model, a three compartment model was also developed to study the hepatobiliary kinetics of [^11^C]erlotinib [144], which combined the hepatocyte compartment and the intrahepatic bile duct compartment into a single compartment of liver tissue (Figure 2(b)). In this model, *k*_3_ described the transfer of radioactivity from the liver to the extrahepatic excreted bile, which can be observed on the PET images and corresponds to the extrahepatic portion of the bile duct combined with the gall bladder [144]. This model has also been successfully applied to describe the hepatobiliary kinetics of [^11^C]tariquidar in humans and mice [84] and the kinetics of [^99m^Tc]mebrofenin in rats [145], demonstrating that it is applicable to different radiotracers and different imaging modalities. Another modeling approach, which included the portal vein contribution to the total input to the liver, was a full PBPK model which included kidney compartments as well as extrahepatic and extrarenal tissue compartments, to which the radiotracer was distributed [146]. This model was applied to whole-body PET data in mice obtained with two experimental radiotracers, targeting the human costimulatory molecule CD80, which predominantly underwent hepatobiliary excretion. Even though a full PBPK model requires more computing capacity and may lead to inaccuracy of the parameter estimates (due to the high number of individual parameters to be estimated), this approach allowed including several physiologically relevant processes to identify alterations in transporter activity at different blood-tissue levels at the same time.

Similar models as those discussed above have been developed to represent the hepatobiliary kinetics of SPECT tracers or MRI contrast agents. For example, some models have been implemented to describe the pharmacokinetics of [^99m^Tc]mebrofenin in the liver of healthy volunteers and patients with liver disease [106,111,112]. However, these models did not consider the contribution of portal vein blood to the blood input for the liver and might therefore not accurately represent the liver uptake clearance. In addition, a similar model to the one developed by Ørntoft et al. for [^11^C]cholylsarcosine PET data [89] was used to evaluate the hepatic distribution of gadoxetate and quantify liver perfusion and hepatocyte function [147]. MRI with contrast agents provides images with higher spatial resolution compared to PET data, making it possible to directly derive both the hepatic artery and the portal vein concentration–time curves from the MR images. This obviates the need of implementing a mathematical model to estimate the portal vein concentration and also reduces the number of model parameters to be estimated. Other, simpler pharmacokinetic models, which also included a dual blood input, have been implemented with gadoxetate in order to quantify liver perfusion [148,149]. In addition, more complex models have been proposed for different contrast agents, including gadoxetate, in order to derive quantitative parameters to assess liver lesions [150] or to evaluate and quantify hepatic perfusion and function [151]. Even though only a few studies have been published so far, in which pharmacokinetic models have been applied to MRI data to study hepatic transporter activity, this topic is currently subject of a research consortium funded by the EU Innovative Medicines Initiative named TRISTAN (Translational Imaging in Drug Safety Assessment, see: https://www.imi-tristan.eu/).

## 6. Challenges in quantitative liver PET imaging

PET in combination with PBPK modeling offers the possibility to quantitatively study the liver pharmacokinetics of a radiotracer and to evaluate hepatic transporter activity. This can potentially be applied in drug development to assess the influence of hepatic transporters on the excretion of radiolabeled drugs, such as in transporter-mediated DDIs. Moreover, this approach bears considerable potential for diagnostic functional liver imaging as hepatic transporter activity changes in liver disease [13,14]. However, it is important to bear in mind that PET has some limitations, which can lead to a misinterpretation of the results. One limitation of PET is the limited spatial resolution (of the order of a few millimeters, see Table 1), which makes it difficult to accurately measure radioactivity concentrations in small structures, such as the portal vein or the intrahepatic bile ducts in the liver, due to the possible occurrence of partial volume effects. A potential solution is the use of hybrid PET/MR scanners, in which the high-resolution anatomical details from MRI can be potentially exploited to localize the portal vein and correct for partial volume effects in PET enabling to extract the input function directly from the PET data. Moreover, the development of dual-modality imaging probes such as ^111^In-EOB-DTPA/Gd-EOB-DTPA, which allow for simultaneous SPECT and MR imaging, may combine the advantages of both imaging modalities. Another limitation of PET imaging with the purpose to study drug disposition is the short half-lives of the radionuclides available for radiolabeling of low-molecular weight drug molecules (e.g. ^11^C, ^18^F). This limits the maximum possible duration of a PET scan to approximately 1.5 h for ^11^C and 5 h for ^18^F, which may not be sufficient to accurately describe the pharmacokinetics of drugs with a low plasma clearance. Moreover, due to dosimetry concerns, PET tracers are rarely administered orally [152], so that some important aspects of drug disposition, in which transporters may play a role (e.g. absorption from the intestine, first-pass extraction in the liver), cannot be assessed. A particular challenge for imaging drug disposition in the liver is the presence of radiolabeled metabolites, as PET cannot distinguish between parent radiotracer and radiolabeled metabolites. This essentially limits the applicability of PET for the study of drug disposition to drugs which do not undergo clearance by metabolism, but which are excreted in unchanged form. However, even for drugs which are excreted in the form of metabolites, the short duration of a PET scan may represent a time window in which metabolism is still negligible, so that some aspects of the hepatic disposition of the parent drug can be assessed. In such scenarios it is, however, questionable whether the PET data accurately reflect the disposition of the drug over a longer time window, during which the emergence of metabolites comes into play. Based on these limitations, it is certainly preferable to employ prototypical transporter substrate radiotracers (Table 2), which have been designed to display optimal characteristics for transporter imaging, including good metabolic stability.

## 7. Conclusion

Noninvasive imaging methods (PET, SPECT, and MRI) in combination with advanced pharmacokinetic models can be applied to quantitatively assess the pharmacokinetics of radiotracers or contrast agents in the liver. This approach can be employed to assess the activity of hepatic transport proteins, which play a crucial role in drug disposition, provided that imaging probes with suitable characteristics for transporter imaging are available. Liver imaging has some potential in drug development, to assess transporter-mediated excretion of novel drug candidates including transporter-mediated DDIs. Moreover, transporter imaging potentially plays an important role in diagnostic functional liver imaging, as alterations in transporter activities occur in various diseases.

## 8. Expert opinion

During drug research and development, it is important to obtain a detailed understanding of the mechanisms involved in the clearance of new molecular entities, since clearance is one of the major determinants of the systemic and tissue exposure to drugs. The inability to precisely quantify and/or predict clearance as well as the concentrations of a drug in the tissue targeted for treatment can have major implications for drug safety and efficacy. Several drug clearance classification systems based on *in vitro* obtained properties of the drugs under development have been proposed to predict the major clearance route of new molecular entities [21–24,153]. Since the liver is the major organ involved in the excretion of drugs from the organism, determination of the total hepatic clearance is a pivotal step in understanding and predicting the total systemic clearance of a drug. Hepatic clearance depends on different processes such as sinusoidal uptake, sinusoidal backflux and canalicular efflux, which are mediated by transporter proteins located in the cellular membrane of hepatocytes. Available *in vitro* systems to obtain parameters that define these processes (e.g. primary hepatocytes) can have limited value due to unsatisfactory IVIVE of these parameters [154,155]. For instance, PBPK models, which implement the *in vitro* obtained parameters, have been proposed in order to predict the transporter-mediated pharmacokinetics of drugs in humans [36,155–158]. These models usually employ scaling factors that may lack the ability to accurately represent differences in transporter abundance, activity, and localization between *in vitro* systems and the *in vivo* situation, potentially leading to the inaccuracy of predicted *in vivo* parameters [155,158–160]. Moreover, even though these approaches may in certain cases be applied to predict the total systemic clearance of a drug, they cannot straightforwardly predict drug concentrations in target or vulnerable tissue [37]. Noninvasive imaging methods, such as PET and SPECT, can be employed to bridge this knowledge gap and directly measure the tissue distribution and pharmacokinetics of radiolabeled drugs in humans. This may be employed to elucidate clearance mechanisms and to obtain information on the influence of transporters on the hepatic disposition and elimination of drugs. Several studies have addressed these questions by employing PET with radiolabeled drugs. Already marketed drugs, such as metformin, telmisartan, rosuvastatin, glyburide or erlotinib, have been studied with PET to assess their interactions with hepatocyte transporters and/or their vulnerability to transporter-mediated DDIs, in combination with prototypical transporter inhibitors [37,56,66,70,77,78,94,97,98]. This approach may potentially also find application in the study of new drug candidates to obtain crucial information on tissue distribution and transporter-mediated clearance, which may ultimately improve drug safety and efficacy. Moreover, the direct *in vivo* measurement of tissue clearances of radiolabeled drugs in humans may help to validate and refine currently available IVIVE approaches by direct comparison of *in vivo* obtained values with values from *in vitro* systems (e.g. sandwich-cultured hepatocytes, transporter-overexpressing cells) [154]. This approach is exemplified by a study, in which a prediction method for hepatobiliary clearances and hepatic concentrations of rosuvastatin based on *in vitro* data in sandwich-cultured rat hepatocytes and in transporter-expressing cell lines, was successfully validated in rats with [^11^C]rosuvastatin PET data [36]. Even though PET imaging appears to be a promising tool for drug research and development, there are certain methodological limitations such as the inability of the PET-obtained radioactivity measurements to differentiate between the radiolabeled parent drug and its metabolites. Therefore, assessment of clearance mechanisms of radiolabeled drugs with PET is essentially limited to drugs which are excreted in unchanged form. For drugs, which undergo extensive metabolism over the duration of the PET scan, the interpretation of the liver PET data will be very complex. Nevertheless, even in such cases some of the PET-derived pharmacokinetic parameters may be incorporated into more complex PBPK models, which take metabolic clearances into account, in order to obtain a comprehensive model of the disposition of the drug under investigation. Due to the limited field of view of currently available clinical PET scanners, kinetic PET data in humans can usually only be obtained for one segment of the body covering an axial length of approximately 20 cm. This does not allow us to measure radiotracer kinetics in several organs at the same time (e.g. liver, intestine, kidneys, and urinary bladder) to provide a comprehensive picture of the whole-body disposition of a radiotracer, which could be quantitatively analyzed with PBPK models. In this context, newly emerging total-body PET scanners, which cover an axial field of view of 200 cm (i.e. the EXPLORER PET scanner) [161,162], offer the unprecedented possibility to obtain kinetic information on the whole-body disposition of radiolabeled drugs with considerably improved sensitivity. This approach may be potentially very useful in providing a better understanding of whole-body disposition of known drugs and drug candidates and may be helpful to unveil complex DDIs which involve several body tissues or organs at the same time. Due to the high costs and complex infrastructure required for PET imaging, this method will most likely not be routinely applied in drug development to assess transporter-mediated drug clearance. Nevertheless, PET imaging has the potential to provide essential mechanistic information on drug pharmacokinetics, which cannot be obtained with any other currently available methodology.

## Article highlights

- Assessment of hepatic transporter activity with imaging methods (PET, SPECT, MRI) requires transporter probe substrates, which need to fulfill certain criteria for effective transporter imaging.
- A comprehensive, up-to-date list of currently available PET and SPECT tracers and MRI contrast agents for imaging hepatic transporter activities is provided and their applications for transporter imaging are discussed.
- Mathematical modeling approaches to extract quantitative values of transporter activities from imaging data are reviewed.
- Methodological limitations of imaging-based assessment of hepatic transporter activities are critically discussed.
- The potential applicability of hepatic transporter imaging in drug development is discussed including the assessment of transporter-mediated drug-–drug interactions and the validation of prediction methods for hepatic clearance of drugs.
